# The Value of Certain Therapeutics in Functional and Organic Nervous Diseases*Read before the Mitchell District Medical Society.

**Published:** 1897-01

**Authors:** Curran Pope

**Affiliations:** Louisville, Ky.; Professor of Diseases of the Mind and Nervous System in the Louisville Medical College; Consulting Neurologist to the Louisville Medical College Hospital


### THE VALUE OF CERTAIN THERAPEUTICS IN FUNCTIONAL AND ORGANIC NERVOUS DISEASES*.

By CURRAN POPE, M.D., LOUISVILLE, KY.

Professor of Diseases of the Mind and Nervous System in the Louisville Medical College; Consulting Neurologist to the Louisville Medical College Hospital.

The vast number of cases of neurasthenia, largely the results of modern modes of living and high pressure work, to-day throng the offices of physicians in search of the "Fountain of Health" from whose crystal waters restoration and relief will flow. Alas, like Ponce de Leon, their futile journey is along the swamps and morasses of failure—nay, even of despair, and fortunate are they whose physicians realize their *ignis fatuus* and turn them from the errors of their ways. The sooner these cases quit following the will-o'-the-wisp in the shape of the mineral water fad, the sooner they shun fashionable watering-places, the sooner they stop swallowing pills, powders and potions, the better will be their lot. And how can relief and cure be obtained? By hard work. The hardest labor a neurasthenic patient ever underwent is the labor of patiently and painstakingly following a line of rational therapeutics. The neurasthenic state is marked by more or less persistent diminution of nervous energy or force with increased susceptibility to external impressions, and involves in its octopus grip the whole nervous and general system; originates from a multiplicity of causes, and possesses its thousands of subjective symptoms. Lately its pathology and pathological anatomy have become well understood and from a rational basis for therapeutics, diminished and weakened nerve energy with great nerve irritability, characterize the disease and underlie nearly all its symptoms. The lack of proper cell nutrition together with the accumulation of fatigue products, a failure to eliminate same, and assimilate appropriate nutritious

*Read before the Mitchell District Medical Society.

pabulum presents a pathological problem full of interest. These cases are all chronic, though there is no danger to life. Really and truly it is little short of marvellous how cures can be wrought out of the material at hand, and though the difficulties are great they are rarely insurmountable. But to reach a perfect result requires plenty of time, great sacrifice on the patient's part, a variety of remedial measures out of the ordinary, and a vigilance that never tires. The patient must be taken from his surroundings, in order to remove and lessen the etiological factors or break up their vicious cycle. They need rest. Not the questionable rest of travel, sea-shore or mountain, but real rest that will permit of the storage of energy, while at the same time remedial measures may be adopted to remove the retained toxic products, or prevent their reformation. It must be rest, partial or total, and this in a sanatorium, where we can regulate their daily life. The bad features of rest can be overcome by the usage of massage, vibration and electricity. This well recognized mode of treatment, so ably worked out by Mitchell, has given me the most satisfactory results, when further amplified by certain hydro-therapeutic measures, such as the dripping sheet, pack, douches, rain baths, etc. Minute attention to details, a corps of specially trained massage operators, nurses and attendants are absolutely essential to the successful carrying out of every feature. This is no place for a description of a mode of treatment so well known; I cannot praise too highly its results in carefully selected cases. The partial rest cure, coupled with a generous diet, hydro-therapy, vibration, exercise and electricity has in a goodly number of cases yielded brilliant results. The hearty co-operation of the patient is essential, and the physician must be master of every move. In hystero-neurasthenia and hysteria itself, its field of usefulness is vastly broadened and its value much greater. In fact, in this psycho-neurosis it frequently is the only method by means of which we can attain a satisfactory result. Nowhere in the range of therapeutics are so many mistakes made as in hysteria. Little attention and indiscriminate prescribing are the usual routine methods of treating these cases, when, in reality, careful, painstaking and systematic use of this valuable mode of treatment will attain results which are brilliant in the extreme, raising the apparently lifeless and hopeless human to a sphere of happiness and usefulness beyond his or her most rosy dream. The necessary equipments are such as to absolutely preclude its application in private homes, or general infirmaries, and many failures are a very natural result. A calm, judicial bearing, the inspiration of confidence, a thorough knowledge of every detail of the case, an intricate knowledge of human nature, the patience of Job, every mechanical, electrical, and hydro-therapeutical appliance is essential to a proper management of these cases. Drugs are valueless and are merely used for passing complications. Hypnotics, sedatives and opiates should not be used.

The constantly increasing experience of neurologists confirm every word written. But there are many neurasthenics and hystero-neurasthenics who cannot place themselves in a sanatorium, but who can take regular office treatment or a partial rest cure. To these a carefully laid out schedule of rest, exercise, diet, hydrotherapy, massage, and electricity will frequently give very satisfactory results. These cases have many of the causative factors still acting, and as a consequence labor under many difficulties. These must be told and taught that persistence in treatment is the only way by means of which they can recover their health and retain it. In these cases we must limit our medicinal treatment to a minimum, and Dercum suggests only two drugs possessing any value whatever, viz.: antipyrin and bromide, both of which must be used with great care only for short periods. Complications must be met, but their treatment should not be such as to overshadow the real treatment of the case. Surgery is practically useless or merely preparative in these cases.

The treatment of epilepsy of functional or organic origin has been the opprobrium of medicine. The bromides, Flechsig's opium-bromide treatment, codeia, borax, belladonna, digitalis, and adonis vernalis are still considered the best drugs. In old long standing cases Flechsig's method for a combination of bromide, codeia, and adonis vernalis has yielded me the best medicinal results. In no class of cases must we search so thoroughly for causative factors, functional and organic. Many cases thought functional are really organic, and cases of idiopathic epilepsy are becoming smaller and smaller. I agree with Peterson that reflex epilepsy is so rare it is a curiosity, and ocular tenotomies, operations for phimosis, removal of the ovaries, etc., are unjustifiable, *as a cure,* but ocular defects, phimosis or purulent ovaries should be removed in order to pave the road for the neurological work. This should consist of careful moral control, exercise and a careful diet ; some occupation, the use of the above remedies, combined with the persistent and unremitting use of hydrotherapy and electricity continued for one or two years. Collins has shown what can be done by persistent treatment, and I could duplicate some of his worst cases with successful results in my own practice. In epilepsy I have, through the above combinations, secured such results, that I feel every case should be given such a trial. The value of mechanical vibration or massage in paralysis is a method that I have used for the last five years with great satisfaction, and am of the opinion that it is far superior to the manual massage in the rapidity and permanence of its results. These cases are usually considered so hopeless that little can be done. This has not been the case in a fair number of instances in my own experience.

The writer offers an apology for the fragmentary nature of this paper, for any one of the subjects lightly touched might form the basis of a number of articles without probing deeply under the surface. He has simply voiced his personal results based on a somewhat extended experience.

---

### TREATMENT OF ANAL PRURITUS.

Try first very warm lotions or irrigations two or three times a day; patient to avoid constipation and always to precede movement by irrigation of oil and to anoint the anus with vaseline. Then try in succession named the following preparations:

Morning and evening a painting of the following glycerole: (*a*) Alum, 4 grams; calomel, 2 grams; glycerine, 20 grams. This pomade: (*b*) Calomel, 4 grams; vaseline, 30 grams. (*c*) Oleate of decocaine, $\frac{1}{20}$ part; lanoline pure, 3 parts; vaseline, olive oil, *āā*, 2 parts. (*d*) Red oxide of mercury, 4 grams; vaseline, 30 grams. (*e*) Introduce into anal orifice a tampon of absorbent cotton soaked in a solution of oxide of zinc to 4 p. 30. (*f*) Cauterize the painful parts with a solution of agnoz, $\frac{1}{10}$. (*g*) In quite rebellious cases turn to linear scarifications or to cauterizations with the galvano-cautery.—MORAIN, *Journal de Médecin de Paris.*—(*Ex.*)

